# Rodent tests of depression and anxiety: Construct validity and translational relevance

**DOI:** 10.3758/s13415-024-01171-2

**Published:** 2024-02-27

**Authors:** Sinem Gencturk, Gunes Unal

**Affiliations:** Behavioral Neuroscience Laboratory, Department of Psychology, Boğaziçi University, 34342 Istanbul, Turkey

**Keywords:** Depression, Behavioral despair, Anxiety, Animal models, Behavioral testing, Cognitive affective bias

## Abstract

Behavioral testing constitutes the primary method to measure the emotional states of nonhuman animals in preclinical research. Emerging as the characteristic tool of the behaviorist school of psychology, behavioral testing of animals, particularly rodents, is employed to understand the complex cognitive and affective symptoms of neuropsychiatric disorders. Following the symptom-based diagnosis model of the DSM, rodent models and tests of depression and anxiety focus on behavioral patterns that resemble the superficial symptoms of these disorders. While these practices provided researchers with a platform to screen novel antidepressant and anxiolytic drug candidates, their construct validity—involving relevant underlying mechanisms—has been questioned. In this review, we present the laboratory procedures used to assess depressive- and anxiety-like behaviors in rats and mice. These include constructs that rely on stress-triggered responses, such as behavioral despair, and those that emerge with nonaversive training, such as cognitive bias. We describe the specific behavioral tests that are used to assess these constructs and discuss the criticisms on their theoretical background. We review specific concerns about the construct validity and translational relevance of individual behavioral tests, outline the limitations of the traditional, symptom-based interpretation, and introduce novel, ethologically relevant frameworks that emphasize simple behavioral patterns. Finally, we explore behavioral monitoring and morphological analysis methods that can be integrated into behavioral testing and discuss how they can enhance the construct validity of these tests.

## Introduction

*“Actions of all kinds, if regularly accompanying any state of the mind, are at once recognized as expressive. These may consist of movements of any part of the body, as the wagging of a dog’s tail, the shrugging of a man’s shoulders, the erection of the hair, the exudation of perspiration, the state of the capillary circulation, laboured breathing, and the use of the vocal or other sound-producing instruments. Even insects express anger, terror, jealousy, and love by their stridulation.”*

Charles Darwin, The Expression of the Emotions in Man and Animals, 1872

**Fig. 1:**
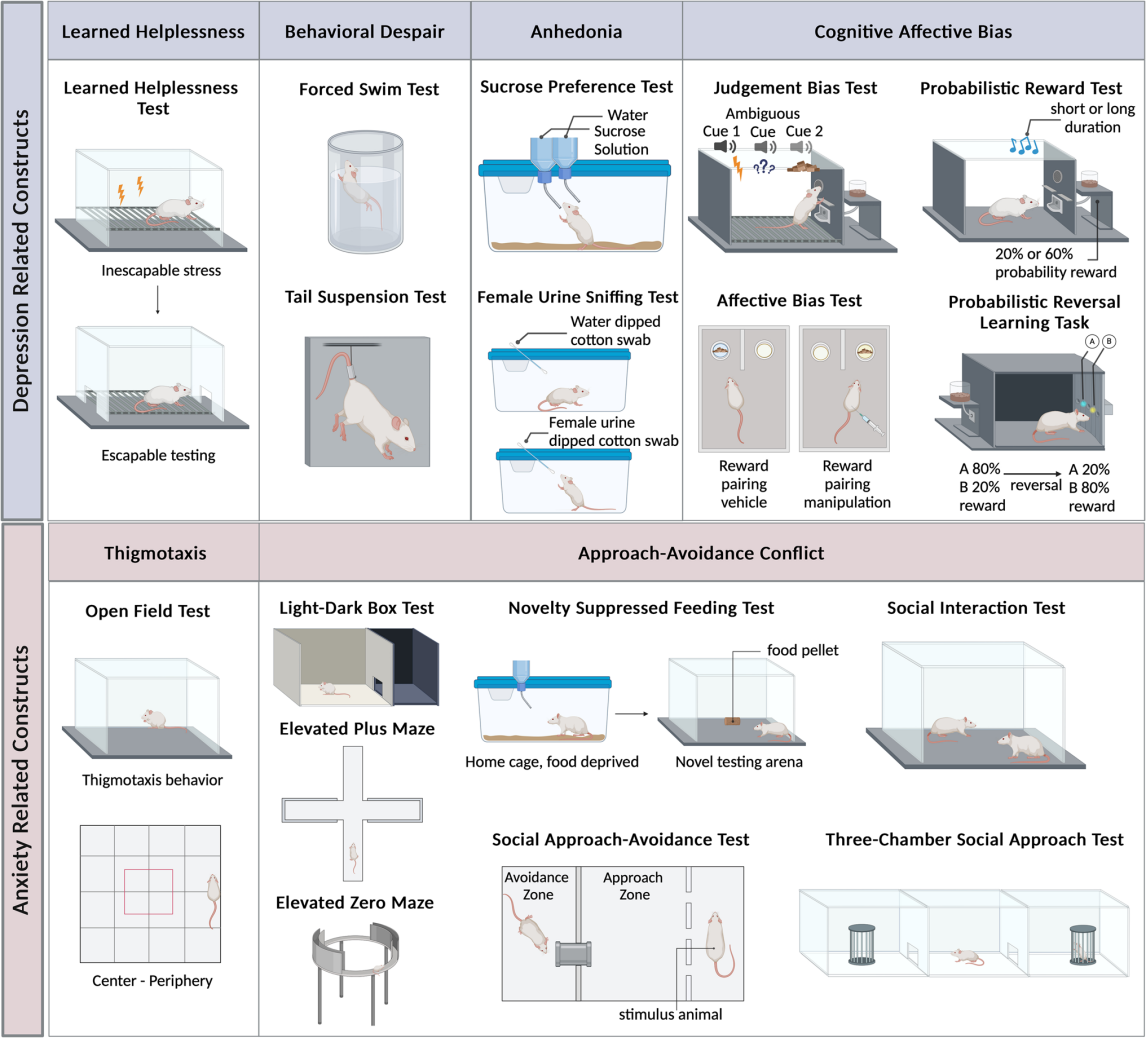
Different depression (top panel) and anxiety-related (bottom panel) behavioral constructs and tests. Images were created with BioRender

Diagnosing clinical depression and anxiety requires a medical interview that covers patient’s health history and reported symptoms. In addition to other clinical observations and tests, the medical history collected through patient self-reports provides key information to determine whether the diagnostic criteria are met. The most common diagnostic classification systems used for this purpose are the DSM-5 (American Psychiatric Association, 2013) and the ICD-10 (World Health Organization, 1992). Preclinical research with rodent models, in contrast, often depend on brief observation of particular species-typical behaviors. These rodent behaviors, which are triggered by either aversive or appetitive stimuli in a custom-purpose maze or chamber, are used as measures that resemble depressive- and anxiety-related states in humans (Belovicova et al., 2017). In addition to preclinical research (i.e., drug development), utilizing rodent constructs of depression and anxiety constitutes an essential component of basic neuroscience studies designed to elucidate their neurobiological underpinnings (Unal & Moustafa, 2020). This review provides an overview of the construct validity of the major rodent tests used to assess behaviors that are hypothesized to resemble certain aspects of clinical depression and anxiety. We start with *behavioral constructs*, or theoretical framework of specific disease-related rodent behavior, and then explain individual *behavioral* *tests* used to assess these constructs (Fig. 1). The first part of each behavioral test provides the most common version of the experimental procedure, whereas subsequent paragraphs focus on discussions of construct validity and translational relevance.

In animal research, a behavioral test involves evaluating a particular species-typical behavior in relation to a behavioral construct (Pollak et al., 2010). A related concept, an experimental *animal model*, conversely, incorporates manipulating biological or environmental factors to prompt specific behaviors or symptoms in the model organism. Although frequently used interchangeably in the literature, there is a distinction between the two terms. Animal models are used to elicit particular behavioral response patterns or to induce symptomatic behaviors that resemble a human disorder. Behavioral tests, in contrast, are designed to capture the readouts of the model. A behavioral test possesses only a dependent variable—the observed outcome of the manipulation; whereas a model has an independent variable, the manipulation, and a dependent variable (Cryan & Slattery, 2007).

Given that capturing all aspects of human disorders in an animal model is not possible, rodent models on depression and anxiety reflect only certain aspects of these disorders based on their pathophysiology (Becker et al., 2021; Gururajan et al., 2019; Planchez et al., 2019). Certain models, such as those that involve genetic modifications (El Yacoubi & Vaugeois, 2007) or hormonal supplementation (Demuyser et al., 2016) emulate the biological aspects of the disorders. Other models manipulate environmental factors for a particular period of time to mimic human disorders in rodents. These environmental manipulations also are used to capture developmental aspects of disorders. For instance, animals that are subjected to maternal separation during infancy are subsequently assessed in adulthood (Tractenberg et al., 2016).

The forced swim test, tail suspension test, open field test, elevated plus maze, light-dark box test, novelty suppressed feeding test, and social approach tests elicit particular behaviors with mild stressors, such as an aqueous environment, highly illuminated novel areas, or the presence of an unfamiliar conspecific. Other tests, such as the sucrose preference test and female urine sniffing test, trigger particular behaviors by using appetitive stimuli. Cognitive affective bias tests either co-utilize aversive and appetitive stimuli or only rely on appetitive stimuli. While behavioral tests trigger specific behaviors, they do it within the test apparatus (i.e., maze) for transiently eliciting the behavior that they intend to measure. Animal models, in contrast, involve behavioral procedures to induce a cognitive or affective *state* that is associated with depression or anxiety. It must be noted that certain behavioral tests, such as learned helplessness, may be considered behavioral models because of their long-term effects that can subsequently be captured with other tests. For instance, the delivery of inescapable shocks in learned helplessness serves as a model to induce depression-like behavior, whereas the subsequent assessment of escape behavior constitutes the test phase. The animal induced with learned helplessness is more likely to exhibit symptomatic behavior in other behavioral tests that evaluate depression or anxiety-like constructs.

The clinical relevance of a disease model or behavioral test is scrutinized under different types of validity including face, predictive, and construct validity (Willner, 1986). Certain types of validity, such as etiological validity, are particularly pertinent for independent variables and commonly considered in behavioral models, whereas others, such as convergent and discriminant validity, hold greater significance for behavioral tests (Becker et al., 2021; Geyer & Markou, 1995; Gururajan et al., 2019). In contrast, construct validity, the central focus of this review, is a fundamental concept applicable across all experimental applications. In a model, it pertains to the efficacy of inducing the desired symptoms in animals (Belzung & Lemoine, 2011). In the context of a behavioral test, construct validity depends on the capacity of the test to effectively measure the intended behavioral construct (Geyer & Markou, 1995).

Clinical depression and anxiety are two distinct, yet highly interconnected mental disorders. Depressive symptoms and anxiety disorder have a high level of comorbidity (Kessler et al., 2015; Lamers et al., 2011). Animal models reflect this close relationship, and there is only a vague distinction between rodent behaviors associated with depression and anxiety (Kalueff et al., 2007). The blurry demarcation between psychiatric disorders may, to a considerable extent, be attributed to the diagnostic criteria employed in clinical practice. These diagnostic criteria are criticized because of the noticeable heterogeneity of symptoms within categorized disorders, as well as the high degree of comorbidity among different disorders (Regier et al., 2009). Relying on higher-order symptomatology for the distinction of psychiatric disorders further obscures the relationship between basic neuroscience and clinical research.

The Research Domain Criteria (RDoC) framework (Sanislow et al., 2010) was developed to overcome the aforementioned challenges and to establish a common theoretical ground between behavioral neuroscience (i.e., animal) research and human psychopathology. This framework aims to deconstruct the complex symptom-like higher-order constructs into simple behavioral patterns and facilitates associating individual symptoms with specific neurobiological mechanisms. Behavioral neuroscientists have long endeavored to establish correlations between behaviors observed in animal testing and symptoms of human disorders. For instance, immobility behavior in the forced swim test is commonly referred as a depression-like behavior (Yankelevitch-Yahav et al., 2015). In fact, it more likely resembles psychomotor retardation observed in depressed patients (Unal & Canbeyli, 2019). However, the foremost criticism regarding the construct validity of behavioral tests focuses on the interpretation of animal behaviors using frameworks designed for clinical assessments in humans. In the subsequent sections of this article, we discuss that behavioral tests that apparently fail to measure the higher-order constructs that they intend to capture, indeed measure lower-order constructs classified by the RDoC. Utilizing this ethologically relevant framework, instead of aiming a one-to-one correlation between animal behaviors and specific symptoms of depression and anxiety disorders, substantially increase the construct validity of the discussed behavioral tests.

Assessing depression and anxiety-related phenomena in rodents is not limited to behavioral testing in an experimental apparatus. It also includes metabolic and physiological measures, such as blood analysis (Pryce et al., 2005; Touma et al., 2008) and temperature monitoring (Belovicova et al., 2017; Bouwknecht & Paylor, 2008), which are outside of the scope of this review. However, alongside conventional behavioral tests, home cage monitoring, and morphological analysis methods that do not require a specific experimental apparatus have increasingly been incorporated into behavioral testing. These novel methods include assessing the posture (Ebbesen & Froemke, 2021), facial expressions (Langford et al., 2010; Perkins et al., 2012), and ultrasonic vocalizations (USV) (Simola & Granon, 2019; Wöhr & Schwarting, 2013) of the animal. These techniques are based on recording and identifying species-typical behaviors and expressions that can be elicited during behavioral testing or emerge naturally in the home cage (Grieco et al., 2021; Klein et al., 2022).

This review starts with constructs that have been associated with clinical depression. We review behavioral tests that rely on learned helplessness, behavioral despair, anhedonia, and cognitive affective bias. We then explore tests of anxiety-like behavior that depend on two species-typical behaviors: thigmotaxis and approach-avoidance behavior. We discuss the nature and evolution of each construct in relation to its behavioral tests and then explain the standardized protocol for each test. We review the major criticisms regarding the construct validity and translational relevance of each procedure and explore the contribution of novel theoretical frameworks and classifications, such as the RDoC. In the final part, we review the use of supplementary behavioral monitoring and morphological assessment methods in assessing affective states of rodents. We discuss how they can be incorporated into behavioral testing and highlight their potential to enhance the construct validity of the conventional methods reviewed.

## Depression-related constructs

### Learned helplessness

Learned helplessness (LH) stands for ceasing attempts to escape after being exposed to a repeated inescapable aversive stimulus or condition (Seligman & Maier, 1967). In this paradigm, once the animals learn that escape from the aversive environment is not possible no matter what they do, they stop escape attempts and appear helpless when an opportunity of escape actually, and visibly, becomes possible. The perceived uncontrollable and inescapable nature of the aversive event is the identifying feature of LH, and it is more important than other characteristics of the event, such as the type or intensity of the aversive stimuli (Maier & Seligman, 1976). Controllable or escapable aversive stimuli do not lead to a learned helplessness effect (Maier, 1984). Initially observed in dogs (Seligman & Maier, 1967), this paradigm was soon tested and observed in mice (Braud et al., 1969), rats (Looney & Cohen, 1972), fish (Padilla et al., 1970), and humans (Table 1) (Fosco & Geer, 1971; Thornton & Jacobs, 1971).

**Table 1:** Behavioral test protocols for rodents and humans

Behavioral test	Original and updated rodent protocols	Similar or analogous Human protocols
Learned helplessness	Rat: Looney & Cohen, 1972; Vollmayr & Henn, 2001 Mouse: Braud et al., 1969; Chourbaji et al., 2005	Fosco & Geer, 1971; Thornton & Jacobs, 1971; Miller & Seligman, 1975
Forced swim test	Rat: Porsolt, 1977b; Yankelevitch-Yahav et al., 2015 Mouse: Castagné et al., 2010	-
Tail suspension test	Rat: Chermat et al., 1986; Castagné et al., 2010 Mouse: Steru et al., 1985; Castagné et al., 2010	-
Sucrose preference test	Rat: Katz, 1982 Mouse: Liu et al., 2018	Amsterdam et al., 1987; Berlin et al., 1998; Dichter et al., 2010
Female urine sniffing test	Rat and Mouse: Malkesman et al., 2010	Bajpai, 2023
Judgement bias test	Rat: Harding et al., 2004 Mouse: Boleij et al., 2012	Berna et al., 2011; Bourke et al., 2010; Lawson et al., 2002; Gebhardt & Mitte, 2014; MacLeod & Cohen, 1993; Neville et al. 2021; Iigaya et al. 2016
Affective bias test	Rat: Stuart et al., 2013; Mouse: Graulich et al., 2016	Harmer, Bhagwagar, et al., 2003a, 2003b; Harmer et al., 2009a, 2009b; Norbury et al., 2007; Roiser et al., 2012
Probabilistic reward test	Rat: Der-Avakian et al., 2013 Mouse: Luc & Kangas, 2023	Pizzagalli et al., 2005, 2008a, 2008b
Probabilistic reversal learning test	Rat: Bari et al., 2010 Mouse: Ineichen et al., 2012	Murphy et al., 2003; Taylor Tavares et al., 2008
Open field test	Rat: Hall & Ballachey, 1932 Mouse: Seibenhener & Wooten, 2015	Gromer et al., 2021; Walz et al., 2016
Light-dark box test	Rat: Bilkei-Gorzó et al., 1998 Mouse: Crawley & Goodwin, 1980; Bourin & Hascoët, 2003	-
Elevated plus/zero maze	Rat: Pellow et al., 1985 Mouse: Komada et al., 2008	Biedermann et al., 2017
Novelty-suppressed feeding test	Rat: Mitchell, 1976; Blasco-Serra et al., 2017 Mouse: Samuels & Hen, 2011	-
Social interaction / Approach–avoidance test	Rat: File & Hyde, 1978; Haller & Bakos, 2002; Wee et al., 1995 Mouse: Landauer & Balster, 1982; Moy et al., 2004	Lange & Pauli, 2019; Wieser et al., 2010

Originally devised as a measure of hopelessness in nonhuman animals (Seligman & Maier, 1967), learned helplessness was later associated with clinical depression based on the similarity between LH and the etiology and symptomology of depression (Seligman, 1972). The *learned helplessness model of depression* was subsequently developed, suggesting that patients with depression perceive reinforcements more response-independent than healthy individuals (Miller & Seligman, 1973). This theory emphasizes a common cognitive distortion underlying LH and depression. Hence, patients diagnosed with clinical depression often exhibit strong or easily elicited learned helplessness (Miller & Seligman, 1975; William et al., 1975).

A series of experiments sought to evaluate the learned helplessness model of depression by examining the shared cognitive impairments in both learned helplessness and clinical depression (Hiroto et al., 1975; Klein et al., 1976; Maier & Seligman, 1976; Miller & Seligman, 1973; 1975). An early attempt to assess the construct validity of learned helplessness as an animal construct of clinical depression was to test its generalizability to different domains. This was done by inducing LH in humans via physical versus cognitive methods. Participants either received a mild inescapable shock or were asked to figure out insoluble anagrams (Hiroto & Seligman, 1975). This experiment showed that LH is not restricted to the experimental paradigm or domain where it was induced, but it is generalized to other tasks and modalities. This observation led the authors to suggest that LH identifies an induced trait, rather than a state (Hiroto & Seligman, 1975), supporting the theory that LH constitutes an underlying etiological factor for human depression (Seligman, 1972). Additional support for considering LH as a valid construct for depression came from another experiment using the same cross-modality helplessness paradigm (Miller & Seligman, 1975). This study showed that depressed participants with no LH training, and healthy participants who were subjected to LH exhibited similar levels of diminished cognitive performance in an anagram solving test. Although this experiment cannot differentiate the common underlying factor behind the diminished performance observed in both groups, it suggested that LH and clinical depression may have affected the same motivational or cognitive mechanism (Klein et al., 1976; Miller & Seligman, 1975).

This theory and the experiments that provide support for it were quickly criticized to be insufficient to fully explain the versatile etiological factors and symptoms of clinical depression (Costello, 1978). It was revealed that depressed individuals, as opposed to perceiving a lack of connection between their actions and consequences, tended to link the consequences to themselves more often than healthy individuals (Rizley, 1978). In addition, the heterogeneous nature of the clinical population suggested that the cognitive dysfunctions associated with learned helplessness may only apply to a subset of patients (Depue & Monroe, 1978).

The original LH model of depression underwent refinements over time. It was suggested that while perceiving an incompatibility between responses and reinforcements induces learned helplessness in humans, attributing the cause of this incompatibility to internal versus external or global versus specific factors influences the duration and severity of the condition (Abramson et al., 1978). While this theory enhances our understanding of the cognitive processes underlying human learned helplessness, it associates depression with top-down cognitive functions, such as beliefs, expectations, and interpretations, which cannot be assessed in rodents. Consequently, initial experiments focused on symptom-based constructs, such as anhedonia and behavioral despair, rather than delving into the cognitive aspects. However, there is a trend suggesting that affection-related cognitive impairments also can arise by bottom-up processes and be measured by using similar tests in humans and other animals. This has led to the development of cognitive affective bias measurements in animals, reintegrating the once-divergent concepts of depression and cognition in animal testing (Robinson & Roiser, 2016) (refer to the Cognitive Affective Bias section).

#### Learned helplessness tests

Testing learned helplessness in animals requires three groups: two control and one experimental group. One of the control groups does not receive any aversive stimulus, whereas the other control group faces a stressful situation from which they can escape. The experimental group experiences the inescapable version of the same stressful situation. This simple experimental design allowed researchers to distinguish the behavioral effects of stress from the controllability of the stress. The procedure starts with a training period, during which the latter two groups are successively exposed to a number of aversive stimuli (Chourbaji et al., 2005). This is followed by a test session, where the escape from the aversive stimulus or environment is made possible for the experimental group. The learned helplessness effect is deemed to be observed when the experimental group exhibits significantly fewer attempts to escape compared with the control group in the test trials (Overmier & Seligman, 1967).

In learned helplessness tests, choosing an effective aversive stimulus and an ethologically appropriate escape behavior for the model organism is crucial (Vollmayr & Gass, 2013). In rodent testing, the traditional and most common aversive stimulus is a mild foot shock (Seligman & Beagley, 1975; Silveira & Joca, 2023). For the escape behavior, rats are trained to turn a wheel (Drugan et al., 1997) or press a lever (refer to Vollmayr & Henn, 2001 for the rat protocol), whereas mice often are trained to shuttle between two compartments of the box (refer to Chourbaji et al., 2005 for the mouse protocol). Female rodents were initially hypothesized to be resistant to the learned helplessness test (Dalla et al., 2007) because of their superior performance in operant conditioning tasks, such as the active avoidance test (Dalla & Shors, 2009), as well as more pronounced active fear responses (Gruene et al., 2015) compared with males. This led to the conclusion that the escape behavior in the test should pose a greater difficulty for females to exhibit a behavioral effect (Hunziker & dos Santos, 2007; Kokras & Dalla, 2014). However, later studies did observe learned helplessness in female rats (Baratta et al., 2018) and mice (Chourbaji et al., 2010) even in classical shuttle box performance measurements. There is still a need for a standardized test protocol to systematically investigate the role of biological sex factors in learned helplessness.

Initially theorized as an etiological factor in human depression (Miller & Seligman, 1973; Seligman, 1972), the learned helplessness became a behavioral test to induce and evaluate depressive-like behavior in rodents and other animals (Seligman & Beagley, 1975; Vollmayr & Gass, 2013). The LH was employed both as a theory of clinical depression in humans and an animal construct, enhancing its face validity. However, given the aforementioned cognitive aspects of LH, testing this behavior in humans and other animals do not necessarily cover the same phenomenon. For this reason, a more parsimonious term, learned aversive uncontrollability (LAU) was proposed for the construct assessed in the learned helplessness test (Pryce et al., 2011). This suggestion aligns with the RDoC framework, wherein helplessness behavior falls under the sustained threat construct. Notably, Maier (1984), one of the developers of the learned helplessness test, theorized that this construct evaluates a generic form of “stress and coping,” where coping denotes an animal's control over a situation. Maier concluded that learned helplessness therefore pertains not only to depression, but it also extends to other stress-related disorders (Maier, 1984). This terminology expands the scope of learned helplessness, moving beyond assessments solely focused on depression to encompass measurements of anxiety-like behavior (Maier & Watkins, 1998).

### Behavioral despair

Similar to learned helplessness, behavioral despair occurs when the animal encounters an inescapable aversive situation. Following an initial effort to escape the aversive context, the rodent normally decreases its (loco)motor activity and becomes more immobile (Unal & Canbeyli, 2019). This increase in immobility, or decrease in motor activity and struggling, within the aversive context is called behavioral despair (Porsolt et al., 1977a, 1977b). Behavioral despair is elicited and measured with two different tests: the forced swim test (FST) and the tail suspension test (TST) (Castagné et al., 2010) (Fig. 1). The FST was the original test used to induce behavioral despair, first in rats (Porsolt et al., 1977b) and then in mice (Porsolt et al., 1977a). It induces despair by placing the animal in a small water-filled cylinder for several minutes. Behavioral despair had been considered a rodent-specific construct until the FST was successfully applied to *Drosophila* (Hibicke & Nichols, 2022; Neckameyer & Bhatt, 2016). In the *Drosophila* protocol, a fly is aspirated into a chamber filled with 0.08% sodium dodecyl sulfate, and its overall immobility is recorded for 5 min (Neckameyer & Bhatt, 2016). Different types of stressors were observed to increase the immobility of the flies in the FST (Araujo et al., 2018; Neckameyer & Nieto-Romero, 2015), whereas chronic administration of the SSRI citalopram decreased it (Hibicke & Nichols, 2022). These convergent results enhance the cross-species validity of the FST. As an alternative to the FST, the TST was developed in mice to assess behavioral despair in a water-free environment (Steru et al., 1985). Here, the animal is exposed to inescapable stress while suspended by its tail. Although adopted to rats (Chermat et al., 1986), tail suspension is painful in adult rats because of their weight, and this procedure should only be employed with mice.

#### Forced swim test

The procedure consists of placing the animal in a small, water-filled cylinder, forcing it to swim and keep its head above the water level (refer to Yankelevitch-Yahav et al., 2015 for a video protocol). The behaviors of the animal within the cylinder are categorized mainly as swimming, struggling, and immobility. Immobility is taken as the main indicator of behavioral despair; the more the animal stays immobile within the inescapable aversive condition, the more it is thought to display behavioral despair (Porsolt et al., 1977a, 1977b). The forced swim test is divided into two parts. The first part serves as an acclimation period, utilized to establish the aversive conditions intended to induce behavioral despair in naïve animals. The second part is the test session, wherein periods of immobility and other behaviors are observed and compared with other groups. Immobility scores of the test session also can be compared to the acclimation or pretest session when the effects of genetic factors or long-term manipulations are investigated (Atesyakar et al., 2020). Under normal, drug-free, conditions, an animal is hypothesized to significantly decrease its activity between pretest and test session. In rats, the FST is composed of two consecutive days; the first day, a 15-min session constitutes the acclimation period. A 5-min test session is conducted 24 hr following the acclimation or pretest day (Porsolt et al., 1977a, 1977b). In mice, the whole procedure takes 6 min on the same day. The initial 2 min corresponds to the acclimation period, and the last 4 min are analyzed and compared between the groups (Yankelevitch-Yahav et al., 2015).

The mouse protocol was developed differently as mice were observed to display sufficient immobility in a shorter period of time than rats (Castagné et al., 2010). In addition, rats are better swimmers than mice and cope better in water-based tasks, while forced swimming appears more stressful for mice, making a single-day procedure safer and more reliable for them (Pollak et al., 2010). These reasons were criticized by some researchers, who stressed that using different protocols for rats and mice is not justified when both protocols aim to assess the same phenomenon: the effects of antidepressant applications (Armario, 2021).

The idea of behavioral despair and the FST emerged with the practical need of assessing the effectiveness and efficacy of antidepressant drugs. Different types of antidepressant drugs (Detke et al., 1995) and environmental manipulations (Bogdanova et al., 2013) were observed to decrease immobility in the test phase of the FST without altering general locomotor activity levels as assessed in the open field test (OFT; see below). This suggested that the drug-induced decrease in immobility was an “antidepressant” effect and did not arise because of metabolic side effects (i.e., increased physical energy). Commonly prescribed antidepressants, such as selective serotonin reuptake inhibitors (SSRIs) (Detke et al., 1995; Rénéric & Lucki, 1998), norepinephrine-dopamine reuptake inhibitors (NDRIs) (Detke et al., 1995; Rénéric & Lucki, 1998), and tricyclic antidepressants (TCAs) (Barros & Ferigolo, 1998; Kitamura et al., 2002), produced this differential result in the FST. Rapid-acting antidepressants, such as ketamine (Akan et al., 2023; Ecevitoglu et al., 2019; Kingir et al., 2023), replicated the antidepressant-like effect. Nonpharmacological antidepressant manipulations, such as environmental enrichment (Guven et al., 2022), also may produce therapeutic effects, providing further support for considering FST as a general test of antidepressant efficacy. Acute (Ünal et al., 2022) or chronic stress models (Kingir et al., 2023), in contrast, worsen behavioral despair in the FST by further increasing immobility compared with control groups. Sensitive to several drugs and applications that are known to have a therapeutic effect in the clinic, the FST emerged as a convenient tool to predict antidepressant efficacy and became the “gold standard” for assessing depressive-like behavior in rodents (Unal & Canbeyli, 2019).

Despite its widespread use in neuroscience, the FST is criticized for not measuring the affective phenomenon known as behavioral despair, but producing differential results because of other factors (Nestler & Hyman, 2010; Molendijk & de Kloet, 2015). Notably, early life adversity and prodepressant drugs do not reliably induce changes in FST behavior, prompting questions about the broader applicability of this method to depression or its specificity to stress-related biology. The early criticisms centered on the idea that immobility is not a sign of despair, but rather reflects an adaptive energy-conservation mechanism. One year after the publication of the original study (Porsolt et al., 1977b), a striking article titled “Swimming Rats and Human Depression” was published, postulating that immobility in the FST is a learned behavior to minimize energy consumption (Hawkins et al., 1978). The authors observed that rats could learn to stand on their tails and hind legs to stay alive without swimming and concluded that immobility was an adaptive response to save energy (Hawkins et al., 1978). It is important to note that the current FST protocols keep the water level at 30 cm for rats (Slattery & Cryan, 2012), instead of the original 15 cm (Porsolt et al., 1977b), and prevent rats from standing.

In a later study, researchers ran a 2-hr-long FST session and divided rats into sinking and nonsinking groups based on whether they sank into the water during the session (Nishimura et al., 1988). They observed that nonsinking rats remained in the water for up to 2 hr by floating, demonstrating immobility, whereas those exhibiting less immobility eventually sank. More importantly, they were able to predict whether a rat would sink based on its immobility level during the first 15 min of the session. Rats that swam or struggled more in the early phase of the test were more likely to sink later. The authors concluded that immobility is an adaptive mechanism to prevent sinking, providing support for the criticism that forced swimming-led immobility is an acquired behavior and not a reflection of a depressive-like state (Nishimura et al., 1988).

Considering forced swimming-induced immobility as an adaptive response led researchers to focus on the relationship between learning processes and the FST. If immobility were an acquired response, early exposure to the test environment would alter it. Accordingly, rats that were familiarized with the FST environment before testing displayed more immobility in the test phase. This effect persisted when the animals were familiarized with an empty (no water) FST cylinder or in a cylinder with 4-cm-high water (Borsini et al., 1986). It is suggested that the “emergency responses” of animals decrease under familiar environments (West, 1990). Familiarization with the test procedure also decreased immobility, which was reversed by administration of anisomycin, a memory-disrupting agent (De Pablo et al., 1989), supporting the idea that immobility in the FST is a learned behavior.

A more recent view considers FST as a test of coping strategy against an acute stressor. According to this theory, the FST does not only respond to depressive-like states, but it also is sensitive to other conditions associated with acute stress, such as the autism spectrum disorder (Commons et al., 2017). Hence, results of the FST should not be overinterpreted in relation to depression but can be used as a generic stress response scale. Stress-induced behavior in the FST starts as active coping (i.e., swimming and struggling) and turns to passive coping, observed as immobility, with adaptation to the situation (de Kloet & Molendijk, 2016; Molendijk & de Kloet, 2015). According to this view, antidepressants decrease immobility in the FST via disrupting learning and the adaptation process of the animal (De Kloet & Molendijk, 2016; De Pablo et al., 1989). This interpretation relies on the observation that practically all antidepressant drugs possess cognitive side effects and alter memory processes. However, it does not explain how nonpharmacological antidepressant manipulations, such as environmental enrichment, prevents behavioral despair. Environmental enrichment has been shown to facilitate learning (Falkenberg et al., 1992; Guven et al., 2022; Schrijver et al., 2002); however, it often leads to a decrease in immobility in the FST. By restricting its argumentation to antidepressant drug use in the FST, the “coping strategy” interpretation found good support in the literature (Molendijk & de Kloet, 2019).

The criticisms of FST pinpoint the difficulty of modeling the complex cognitive and affective symptoms of clinical depression in a simple design. The characterization of behavioral despair as a depressive-like symptom was criticized to be an overinterpretation, because the observed immobility in the FST also could reflect a learned behavior or an adapted acute stress response (De Kloet & Molendijk, 2016; De Pablo et al., 1989). However, the FST has proven to be a useful, and often reliable, method to detect the effects of several different antidepressant interventions (Bogdanova et al., 2013; Petit-Demouliere et al., 2005). Based on these observations, it was argued that the immobility in the FST might be more connected to the low-level, sensorimotor symptoms of depression, rather than its high-level cognitive or affective aspects (Canbeyli, 2010). Psychomotor retardation is one of the core symptoms of severe depression, which also can be assessed in nonhuman animals (Willner, 1990). Development of the immobility response in the FST can be considered as a low-level indicator of depression that mimics psychomotor retardation (Unal & Canbeyli, 2019). Psychomotor alterations in rodents can be evaluated in a stress-free way by using home cage monitoring systems (Fureix et al., 2022) (refer to the Home Cage Monitoring section). Importantly, it was observed that the inactive but awake state in the home cage predicted immobility in the FST (Maclellan et al., 2022).

#### Tail suspension test

Unlike the forced swim test, the tail suspension test (TST) was invented to assess behavioral despair in mice (Steru et al., 1985), and later adopted to rats (Chermat et al., 1986). Similar to the 6-min mouse FST protocol, a TST takes 6 min, during which mice are suspended by their tail with a hook (refer to Can et al., 2012 for a video protocol). Movements of the animals and struggling are categorized as searching-behavior, whereas waiting-behavior refers to periods spent immobile (Steru et al., 1985). As in the FST, the antidepressants decrease immobility in the TST (Cryan et al., 2005). This test offers a seemingly less stressful alternative to the FST and abolishes the risk of hypothermia (Thierry et al., 1986), but it is not suitable for adult rats because of their weight.

Because both tests were designed to measure the same construct (i.e., behavioral despair), the TST shares many of the criticisms directed at the FST (Nestler et al., 2002). Accordingly, immobility observed in the TST also may reflect a learned, adaptive strategy or passive coping to an acute stressor. In fact, in both tests, behavioral despair is induced with an acute stressor. Human depression, in contrast, develops with time through combination of several etiological factors. The acute nature of behavioral despair designs does not reflect the developmental pattern of depression (Nestler & Hyman, 2010). Furthermore, while the TST utilizes a more ecologically relevant procedure to induce behavioral despair compared with the water-based FST, it still relies on an artificial stressor, as being suspended by the tail is an unlikely event for rodents. Utilizing a more natural stressor would increase the construct validity of animal models that assess rodent endophenotypes of neuropsychiatric disorders. Using predatory threat, for instance, is the most efficient method to mimic posttraumatic stress disorder (PTSD) in rodents (Goswami et al., 2013).

Another criticism focuses on the differences in onset of action and the time course of drug effects. Classical antidepressants, such as SSRIs and TCAs, rapidly produce an effect in the FST and TST, while they require chronic treatment to ameliorate depressive symptoms in humans (Cryan & Holmes, 2005; Nestler & Hyman, 2010). This comparison suggests that there are important differences between the neurobiological correlates of behavioral despair and human depression (Unal & Moustafa, 2020). At the behavioral level, however, it is important to note that the antidepressant effect in humans is preceded by certain indicators. Differences in social cue processing (Harmer, Bhagwagar et al., 2003a, 2003b) and emotional bias (Harmer, Hill et al., 2003a, 2003b) are observed before the common ameliorative effects of antidepressants. It can be argued that increased mobility in the FST and TST is a similar early indicator of antidepressant action. The *cognitive neuropsychological model of depression* explains the anticipated impacts of antidepressants assessed through cognitive affective bias tests (refer to the dedicated section). To determine if motor changes in FST and TST reflect these effects, a comparative analysis with cognitive affective bias (CAB) tests could be beneficial. Despite one study that reported no correlation between FST results and CAB (Aliphon et al., 2022), further research is required to investigate this relationship.

### Anhedonia

Anhedonia, or the inability to experience pleasure, is one of the two decisive symptoms to diagnose major depressive disorder (American Psychiatric Association, 2013). Similar to many other symptoms of depression, anhedonia is not a unidimensional phenomenon that can be assessed in a simple construct. In rodents, assessing anhedonia involves measuring responses to naturally rewarding stimuli, such as sucrose. It is related to disruptions in the reward system, which encompasses circuits that regulate emotions (liking), motivation (wanting), and learning (Berridge & Robinson, 2003). However, pinpointing the specific neural dysfunction underlying anhedonic behavior has been a challenging task in neuroscience (Scheggi et al., 2018). It was argued that a generic definition of anhedonia lacks discrimination between reductions in consummatory behavior and alterations in the motivational aspects of behavior (Treadway & Zald, 2011).

The heterogenous nature of anhedonia contributes to the difficulty of identifying the underlying neurobiological foundations of it (Berridge, 1996; Treadway & Zald, 2011). These concerns eventually led to a distinction between consummatory anhedonia, reflecting a reduction in hedonic response or liking, and motivational anhedonia, representing a diminished desire in obtaining or wanting the reward (Treadway & Zald, 2011). The most common tests of anhedonia, the sucrose preference test (SPT) and the female urine sniffing test (FUST), are associated with both the consummatory anhedonia (liking) and motivational anhedonia (wanting) (Markov, 2022).

The consummatory dimension of anhedonia, or liking, can be evaluated by observing facial movements, particularly those involving orofacial muscles (Berridge & Robinson, 2003). The facial response to pleasurable stimuli is evolutionarily conserved, showing homologous patterns across diverse species, including humans and rodents. Both species exhibit similar behaviors, such as tongue protrusions or lip-licking, in response to stimuli associated with sweet taste (Berridge, 2000). Quantifying lip-lick occurrences within a rhythmic licking cluster (i.e., lick cluster size) provides an objective and standardized metric for assessing hedonic liking in rodents (Dwyer, 2012).

For assessing motivational anhedonia, researchers commonly utilize the progressive ratio test and the effort-related choice task and evaluate reward motivation and effort-related decision-making (Der-Avakian et al., 2016; Der-Avakian & Pizzagalli, 2018; Scheggi et al., 2018). The progressive ratio test involves animals engaging in progressively challenging tasks to obtain a reward (Hodos, 1961). The effort-related choice tasks, in contrast, present animals with a decision between a low-effort task offering a low reward and a high-effort task with a more substantial reward (Salamone et al., 1991). These tests require prior training and are preferred to specifically measure constructs related to motivation. Other behavioral tests, such as the affective bias test and the probabilistic reward learning test (refer to the Cognitive Affective Bias section), attempt to evaluate the cognitive processes associated with reinforcement learning.

Unlike behavioral despair, induction of anhedonia requires additional behavioral procedures. Researchers typically induce anhedonia by chronic stress models (Scheggi et al., 2018) and assess it with the sucrose preference test or female urine sniffing test (Fig. 1). The chronic unpredictable mild stress (CUMS) (Burstein & Doron, 2018; Wiborg, 2013) is one of the most prevalent models to induce anhedonia in rodents (Willner et al., 1992; Willner, 2017). For several weeks, the animals experience a variety of mildly aversive stimuli and conditions, such as wet bedding or cage tilting (refer to Kingir et al., 2023 for an example). The stress applications follow a random, unpredictable order to prevent behavioral adaptation of the animals. Other models used to decrease reward-seeking behavior—or create anhedonia—in rodents include chronic restraint stress (Mao et al., 2022), social defeat stress (Riga et al., 2015), and social isolation stress (Brenes et al., 2020; Unal, 2021). All of these models rely on creating a chronic stress environment that resembles the long-term stress exposure that patients with anhedonia may suffer (Esch et al., 2002). The ecological validity of these models is usually considered to be higher compared with learned helplessness and behavioral despair tests, which also can be used to induce depressive-like behavior (Nestler & Hyman, 2010). However, specific models of depression, such as early life adversity, may not consistently produce an effect on reward-seeking behavior in the SPT (Robinson, 2018).

#### Sucrose preference test

The sucrose preference test originates from the observation that chronically stressed rats significantly reduce their consumption of sucrose and saccharin-containing solutions compared with control animals (Katz, 1982). The term preference was added to the test when Willner et al. (1987) modified the protocol to include not one but two bottles: one for the sugar-containing water, and the other for regular water. This study replicated the original observation and revealed that chronic stress exposure reduces preference of the sugar-containing solution. This behavioral change was conceptualized as anhedonia based on the rationale that it reflected decreased reward sensitivity following the CUMS protocol (Willner, 1997). The standard protocol of sucrose preference test involves providing rodents with two water bottles: one with regular, pure water, and the other containing 1–2% sucrose, or a similar sweet substance (refer to Liu et al., 2018 for the protocol). The relative consumption of the sucrose solution to the regular water is measured to assess anhedonia. Beacuse rodents have a natural preference for sweet foods and drinks, the amount of sucrose consumption is negatively correlated with anhedonia. Sucrose consumption is typically measured as a percentage of total liquid (sucrose solution + regular water) consumption to rule out the effects of potential differences in water intake levels because of metabolic factors, such as the weight of the animal (Liu et al., 2018).

Behavioral models of anhedonia rely on natural, or unconditioned, stressors, whereas tests of anhedonia assess species-typical hedonic behaviors. These two characteristics increase the face validity of the construct. However, it is not clear what part of the hedonic response is affected by the anhedonic manipulation, whether the liking or the wanting aspect, limiting the construct validity of rodent tests of anhedonia. In line with this issue, anhedonic behavior is categorized under the “loss” construct in RDoC, which comprises both the behavioral and motivational aspects of anhedonia.

The SPT was criticized for solely assessing consummatory anhedonia related to the liking aspect, measuring suppressed pleasure of consuming a rewarding substance (i.e., sugar) and a subsequent reduction in the preference of that rewarding stimulus (Scheggi et al., 2018). This view proposes to call the test sucrose consumption test. The underlying assumption states that a diminished preference for a sweet solution indicates a decrease in consummatory pleasure. However, it is important to recognize that the SPT is not solely indicative of consummatory behavior, but it also can be influenced by motivational factors as animals actively choose to approach and consume the sucrose solution (Markov, 2022). In addition, the two sides of anhedonia, the loss of pleasure and the loss of motivation have different neurobiological foundations (Berridge, 1996), and anhedonic behavior in rodents have been associated with both circuits (Kingir et al., 2023). The interpretation of the results of SPT may therefore reflect both consummatory and motivational aspects of anhedonia.

Aside from the issues related to defining the type of anhedonia being measured, the SPT encounters additional challenges that impact its construct validity. The test protocol is susceptible to various extraneous variables, such as the timing of the test, the specific animal strain used, and the concentration of the sucrose solution. These factors are acknowledged contributors to the inconsistent results observed in the literature (Berrio et al., 2023; Strekalova, 2023). Confounding variables that systematically differ between the experimental and control groups may arise because of the chronic stress manipulation applied before testing. A recent review article highlighted the importance of correcting for variables, such as the total amount of fluid consumed, the weight of the animal, and the caloric content of the solution. Notably, this correction resulted in a reduction of the observed effects in the SPT (Berrio et al., 2023). Given that chronic stress models have been shown to influence the appetite and body weight of animals (Cox et al., 2011; Willner, 2017), it is crucial to exercise caution when interpreting sucrose preference test results. These results may reflect not only anhedonia but also potential metabolic effects.

In addition, the SPT uses a primary reinforcer, sugar, to initiate and assess reward-seeking behavior, whereas anhedonia in humans often is associated and tested with secondary reinforcers, such as social and monetary rewards (Fussner et al., 2018). Importantly, no change in reward consumption was observed when a similar test was applied to human participants (Table 1) (Amsterdam et al., 1987; Berlin et al., 1998; Dichter et al., 2010). The sweet taste test for humans assesses the pleasantness of liquids with varying sugar concentrations without incorporating a motivational element (Dichter et al., 2010). This human test is primarily designed to measure the hedonic response or liking aspect of anhedonia, sharing similarities with hedonic response measures from facial muscles in rodents (Dwyer, 2012). In contrast, the SPT involves animals freely consuming their choices over a specific period, capturing both the liking and wanting aspects of anhedonia. This methodological difference may account for the observed discrepancies between rodent and human studies. In order to facilitate meaningful comparisons between rodents and humans, it is essential to establish an SPT protocol for humans that closely mirrors the motivational component tested in rodents.

#### Female urine sniffing test

The female urine sniffing test (FUST) (Malkesman et al., 2010) follows a similar procedure with the above sucrose-seeking paradigm; the major difference is the utilized sensory modality. The SPT measures anhedonia through gustation, while the FUST utilizes olfactory stimuli. Two key observations led to the usage of urine to assess reward-seeking behavior in rodents (Malkesman et al., 2010). The initial observation arose from the effective use of urine in olfactory habituation-dishabituation tests (Gregg & Thiessen, 1981). The second observation emphasized the role of pheromone rewards in the sexual behavior of rodents, with the opposite sex's urine serving as a stimulus for male rats (Martínez-García et al., 2009).

The FUST starts with familiarizing male rodents to cotton-tipped applicators in their home cage. Individually housed male rodents are then presented with applicators dipped in water for 3 min to record their total sniffing duration. The urine of female rodents is collected in the estrus phase with cotton applicators, which are inserted into the male cages 45 min after their exposure to the water-dipped applicators (Malkesman, 2011). Different from the SPT, the two options are not made simultaneously available but given consecutively because of their volatile nature. The assessment involves measuring and comparing the duration of sniffing water and female urine across different groups. A reduced duration of sniffing female urine is considered an indicator of anhedonia.

Chemosignals provide different types of vital information for all vertebrates (Brennan & Zufall, 2006). Rodents heavily rely on their olfactory senses to detect and escape predators (Takahashi et al., 2005), locate food (Barnett, 1963), and find potential mates (Kelliher & Wersinger, 2009). As the dominant sensory modality of rodents (Brennan & Keverne, 2004), utilizing olfaction in behavioral testing enhances its ecological relevance. However, as emphasized by the developers of the test, the animal models used to simulate mood disorders can also result in altered olfactory system or gonadal hormone system functioning (Malkesman et al., 2010). This could be a confounding variable in assessing anhedonia-like rodent behavior by presentations of minimal amounts of urine. Additionally, sequential presentation of water and urine prevents the measurement of animal preference when introducing both stimuli simultaneously.

The urine sniffing test was developed a male-only behavioral paradigm. Until recently, behavioral testing in neuroscience was conducted almost exclusively with male rodents, considering that the estrous cycle could introduce confounds in behavioral results gathered from female animals (Beery & Zucker, 2011). Recent research has demonstrated that female animals do not exhibit significantly different levels of variability in the measured constructs across their various hormonal stages (Becker et al., 2016; Prendergast et al., 2014). While female mice exhibit a preference for sniffing the urine of intact male mice over castrated ones (Jemiolo et al., 1985), there is currently no research that employs urine sniffing tests as a measure of anhedonia for female rodents.

Although humans are primarily visual creatures, olfaction plays a critical role in essential functions, such as tracking scents to locate food (Porter et al., 2007) and selecting mating partners (Wedekind et al., 1995). A reciprocal relationship between olfaction and depression in humans has been identified, showing that depressed patients have worse olfaction, while the severity of depression symptoms are positively correlated with olfactory abnormalities (Kohli et al., 2016; Sabiniewicz et al., 2022). Recently, a condensed version of the University of Pennsylvania Smell Identification Test (UPSIT) was created to distinguish individuals with depression based on their reduced smell differentiation scores (Table 1) (Bajpai, 2023). While these studies highlight the significance of the sense of smell in depression and open the door for its application in animal models, establishing a direct link between reduced rodent urine sniffing and anhedonia is not easy. In addition to reducing the incentive salience of naturally rewarding stimuli, depression models may interfere with the sensation of the stimuli itself. As a result, the female urine sniffing test could be interpreted in relation to a physiological symptom of depression rather than directly reflecting anhedonia.

### Cognitive affective bias

The relationship between mood and cognition has been a major theme in the cognitive theories of clinical depression (Copeland, 1970; LeMoult & Gotlib, 2019). In his *cognitive theory of depression*, Beck (1967) heavily emphasized the negative interpretation and appraisal of life events on the development and maintenance of depressive symptoms. The focus on top-down cognitive processes also manifested itself in the reformulated learned helplessness theory of depression (Abramson et al., 1978) (refer to the Learned Helplessness section). More recent attempts concentrated on associating top-down cognitive processes with bottom-up affective (dys)functions (Godlewska, 2019; Robinson & Roiser, 2016; Roiser et al., 2012; Roiser & Sahakian, 2013). The *cognitive neuropsychological model* *of depression* posits that disturbances in monoamine transmission result in bottom-up biases, giving rise to negative perceptions (Roiser et al., 2012). These negative perceptions, in turn, contribute to the formation of dysfunctional negative schemata, which subsequently generate top-down biases, fostering negative expectations. By incorporating disruptions in bottom-up processes, this theory encourages the exploration of cognitive-affective biases observed in human depression through animal testing (Robinson & Roiser, 2016). Moreover, it provides an explanation for the delayed onset of action observed in typical antidepressants, positing that they function not as direct mood enhancers but as agents that initially ameliorate affective processing (Godlewska & Harmer, 2021; Harmer et al., 2009a, 2009b).

Cognitive affective biases (CAB) refer to the biased mental functions, such as attention, explicit memory, and decision-making, that emerge due to an underlying affective state (Hales et al., 2014). The reciprocal relationship between affect and cognition (LeMoult & Gotlib, 2019; Storbeck & Clore, 2007) as well as how this relationship is biased in mood disorders (Deldin et al., 2001; Elliott et al., 2011; Gotlib & Joormann, 2010; Leppänen, 2006) is well-established in humans. Experimental studies showed that depressed individuals interpret ambiguous stimuli in a negative way (Table 1) (Berna et al., 2011; Bourke et al., 2010; Lawson et al., 2002). Similarly, anxiety-prone individuals interpret ambiguous stimuli as worrying (Gebhardt & Mitte, 2014; MacLeod & Cohen, 1993) and miscalculate risk prediction in a negative way (Butler & Mathews, 1983). Building on human studies, it has been suggested that while the majority of cognitive aspects of emotion traditionally involve language-based tasks in humans, many of these tasks could be adapted for examination in animals with appropriate modifications (Paul et al., 2005). Employing a reverse translational approach, the judgment bias test (Harding et al., 2004), which focuses on ambiguous cue interpretation, as well as the affective bias test (Stuart et al., 2013) and the probabilistic reward tasks (Bari et al., 2010; Der-Avakian et al., 2013), which are based on reward processing, were designed to assess cognitive affective bias in animals.

Behavioral tests that assess cognitive affective bias differ from those measuring behavioral despair and anhedonia in two key aspects. First, behavioral despair and anhedonia tests focus on observing behaviors indicative of depression symptoms, whereas CAB tests center around measuring mental distortions theorized to be involved in the etiology and persistence of these mood disorders (Elliott et al., 2011). For this reason, cognitive affective bias measurements, reflecting the affective state of the animal (Paul et al., 2005; Roelofs et al., 2016), also are utilized in relation to anxiety (Burman et al., 2009). The second distinction between cognitive affective bias and symptom-based constructs is the utilization of CAB tests in evaluating the overall well-being of animals (Baciadonna & McElligott, 2015; Bethell, 2015; Boissy et al., 2007; Poirier et al., 2019). A cognitive affective bias can be observed in two directions: positive or negative (Hales et al., 2014). The bi-directional nature of the construct allows researchers to evaluate not only negative behavioral features but also positive affective states in rodents (Paul et al., 2005).

#### Judgement bias test

The first cognitive affective bias assessment method in rodents was the judgment bias test (JBT) developed by Harding and colleagues (2004) (Fig. 1). In this study, experimenters trained rats to press a lever to receive a reward when a certain auditory stimulus was presented. Another auditory stimulus signaled a brief white noise, which would be avoided by not pressing the lever. This constitutes a typical discrimination learning design (Spence, 1936). Rats that successfully completed the training were tested by presenting a novel auditory stimulus with a frequency that falls between the two frequencies of the training stimuli. Animals that pressed the lever were judged to have interpreted the novel stimulus positively, whereas those that did not were judged to have interpreted it negatively. The validity of this construct was tested with animals living under unpredictable cage conditions, which exhibited more “pessimistic” behaviors compared with control animals. It was concluded that the test was able to reflect the mood of animals by showing the effects of aversive stimuli on their cognition (Harding et al., 2004).

Different test protocols were developed after the first study (Bethell, 2015; Boleij et al., 2012). The original protocol (Harding et al., 2004) was conceptualized as a go/no-go paradigm with a reward and a punishment (Roelofs et al., 2016). This procedure requires performing one action to receive the reward and not performing the same action to avoid punishment. If the animal is trained to perform another motor action to avoid the punishment, it becomes a go/go, or active choice, design (Enkel et al., 2010). There are other protocols that omit punishment and utilize a neutral stimulus or two rewards with different value. These reward-reward designs contribute to animal welfare, while eliminating the risk of inducing “pessimism” by the intensity of the punishment (Hales et al., 2014). The go/go designs are deemed more reliable procedures, because it is difficult to interpret the meaning of immobility or lack of action in go/no-go designs (Nguyen et al., 2020). Not displaying an action following the ambiguous cue in the test phase can be intentional or reflect the absence of any behavior (Roelofs et al., 2016).

The JBT is used to assess both depression-like (Enkel et al., 2010; Hales et al., 2014) and anxiety-like states in rodents (Brydges et al., 2012; Burman et al., 2009), as it is considered to reflect affective valence—the positivity or negativity of an animal’s affective state. The JBT is sensitive to both (anti)depressant and anxiolytic/anxiogenic pharmacological (refer to Neville et al., 2020 for a review and meta-analysis) and environmental (Lagisz et al., 2020) manipulations. It also was suggested that a detailed analysis of the behaviors in the test can be used to differentiate anxiety and depression-like phenotypes (Bethell, 2015). Anxiety is associated with increased anticipation of negative events, whereas depression is additionally associated with decreased anticipation of positive events (Eysenck et al., 2006). However, the predictive validity of the JBT for antidepressants is disputed (Anderson et al., 2013), and this test appears to be more sensitive in detecting the negative effects of depressants and anxiogenics than the therapeutic effects of antidepressant and anxiolytic drugs (Neville et al., 2020). Hence, when conducting studies to explore the impact of antidepressants and anxiolytics, it is advisable to either employ larger sample sizes (Neville et al., 2020) or incorporate the recently developed affective bias test (Stuart et al., 2013).

Human experiments that utilize ambiguous stimuli to investigate attitudes and traits on optimism-pessimism resemble the cognitive affective bias. This similarity suggests a strong translation relevance for the JBT, as optimism-pessimism scales are hypothesized to measure human-specific attitudes associated with mood and anxiety (Dember et al., 1989). In addition, unlike other rodent models, the JBT has been applied to a wide range of species, such as bees (Bateson et al., 2011), dogs (Karagiannis et al., 2015), and sheep (Doyle et al., 2011). Also applicable to reptiles and fish (Bethell, 2015), the JBT facilitates comparative research in affect and cognition. Importantly, cognitive affective bias studies in humans following similar protocols to rodent experiments yielded comparable results (Anderson et al., 2012; Iigaya et al., 2016; Mendl et al., 2006; Schick et al., 2013). While earlier human studies utilized secondary reinforcers, such as monetary rewards, a recent study (Neville et al., 2021) directly applied the animal JBT to humans, employing food as the primary reinforcer, and uncovered an association between positive biases and positive affect in humans. These findings led to the consideration of the judgement bias test as the “gold standard” to assess affective states in nonhuman animals (Bateson & Nettle, 2015).

#### Affective bias test

The affective bias test (ABT) (rat protocol: Stuart et al., 2013; mouse protocol: Graulich et al., 2016) was developed to focus on biases in reward-related learning and memory (Robinson & Roiser, 2016). Here, rodents are trained to associate two different cues with a reward. One of the cues is associated with the reward under neutral conditions, while the other reward-pairing is done under a pharmacological or environmental manipulation that alters the affective state. Subsequently, the animals are tested to display their cue preference. The cognitive affective bias is assessed by considering the number of animal choices in the treatment-paired versus control-paired cues. In contrast to the JBT, which focuses on decision making in response to ambiguous stimuli (Roelofs et al., 2016), the ABT adopts a within-subject design that involves comparing the preferences of each animal within their test trials. This approach is grounded in the assumption and observation that animals do not demonstrate bias when both cues are linked to a reward under neutral conditions.

The ABT is responsive to the acute effects of pharmacological or environmental manipulations, as they are administered either immediately before or during the cue-reward association training (Stuart et al., 2013, Robinson & Roiser, 2016). This aligns with the cognitive neuropsychological approach to antidepressant actions, which proposes an initial improvement in cognitive-affective biases, and a subsequent amelioration in mood (Godlewska, 2019; Godlewska & Harmer, 2021; Harmer, Hill et al., 2003a, 2003b; Harmer & Cowen, 2013). Research on both healthy and depressed participants has revealed positive behavioral changes in cognitive-affective biases following acute antidepressant treatment, occurring prior to observable improvements in mood (Table 1) (Harmer, Bhagwagar et al., 2003a, 2003b; Harmer et al., 2009a, 2009b; Norbury et al., 2007; Roiser et al., 2012). The sensitivity of the ABT to acute manipulations also was utilized to differentiate the rapid-acting antidepressant ketamine and the delayed-onset SNRI venlafaxine (Stuart et al., 2015), establishing this test as a valuable tool for studying the mechanisms of action of different antidepressants. Furthermore, an early life adversity model that involved 14 days of postnatal maternal separation (180 min/day) in rats resulted in an impairment in the ABT (Stuart et al., 2019). This suggests that the sensitivity of the ABT is not limited to acute manipulations but also may also extend to certain forms of subchronic or chronic applications.

#### Probabilistic reward test and probabilistic reversal learning task

Alterations in reward processing are strongly linked to clinical depression (refer to the Anhedonia section), and disturbances in reward circuitry are correlated with cognitive function deficits of the disease (Gong et al., 2017). Building on these associations, the probabilistic reward test (PRT) (Der-Avakian et al., 2013) was developed to assess responsiveness to rewards by examining response bias, while the probabilistic reversal learning (PRL) task (Bari et al., 2010) measures sensitivity to positive and negative feedback within the framework of reward learning. Both tests were designed using a reverse translational approach, for which methodologies previously employed in human experiments (Table 1) (PRT: Pizzagalli et al., 2005, 2008a, 2008b; PRL: Murphy et al., 2003; Taylor Tavares et al., 2008) were adapted for rodent testing.

The rationale of the PRT is based on a signal-detection approach (Pizzagalli et al., 2005). In psychology, the signal detection theory is employed to analyze participants’ decisions in uncertain situations (Lynn & Barrett, 2014). It assesses both the sensitivity (the capability to differentiate between signal and noise) and response bias (the tendency to categorize input as either signal or noise) (Stanislaw & Todorov, 1999). Following this methodology, the human task was originally devised (Pizzagalli et al., 2005), and subsequently, the rodent counterpart was developed (Der-Avakian et al., 2013). The human PRT assessed response bias emerging between two similar stimuli when one of them is rewarded more frequently. Participants are expected to adapt their response criteria toward stimuli linked with higher rewards, and the absence of such adjustment is theorized to suggest reduced reward responsiveness (Pizzagalli et al., 2008a, 2008b). Supporting this hypothesis, studies involving depressed individuals have demonstrated a reduced response bias toward stimuli that are frequently rewarded (Pizzagalli et al., 2005, 2008a, 2008b). In rodent experiments, response biases have been effectively induced using acoustic stimuli with a lever-press task (Der-Avakian et al., 2013) and visual stimuli with a touchscreen task (Iturra-Mena et al., 2023; Kangas et al., 2020; Luc & Kangas, 2023), both of which closely resemble the human experiments. Furthermore, as observed in depressed patients (Dillon et al., 2014; Pizzagalli, Evins, et al., 2008a, 2008b), dopaminergic (Der-Avakian et al., 2013) and cholinergic manipulations (Kangas et al., 2020) influence the response bias in rats.

While the PRT measures modulation of the response bias based on a reinforcement history (Der-Avakian et al., 2013), the probabilistic reversal learning task assesses the cognitive flexibility of subjects in adjusting their responses when the probabilistic reward frequency of two previously learned stimuli changes (Bari et al., 2010). In the rodent PRT, rats are trained to poke a hole when illuminated to receive a food reward. Following this, they learn that when two holes are illuminated simultaneously, one of them is more consistently rewarded. The rats are subsequently evaluated for their reversal learning abilities by reversing the reinforcement probability of the stimuli.

Applying the same rationale in humans (Table 1) (Murphy et al., 2003; Taylor Tavares et al., 2008), the PRL task has identified hypersensitivity to negative feedback as a characteristic trait in patients with depression (Elliott et al., 1997; Murphy et al., 2003; Taylor Tavares et al., 2008). Consistent with human studies (Chamberlain et al., 2006), manipulations of serotonin levels induced distinct alterations in sensitivity to negative and positive feedback in rats (Bari et al., 2010) and mice (Ineichen et al., 2012; Phillips et al., 2018). However, studies comparing conventional antidepressants that do not act through the serotoninergic system as well as the rapid-acting antidepressant ketamine yielded conflicting results (Rychlik et al., 2017; Wilkinson et al., 2020). This suggests that the PRL protocol is sensitive to a construct solely related to the serotoninergic system. The task’s relative difficulty for rodents also may contribute to its limited sensitivity to non-serotonergic manipulations. In response to this concern, a novel protocol has been introduced, featuring the separation of discrimination and reversal learning over 2 days (Metha et al., 2020). This design not only improves success in reversal learning for mice but also distinguishes between probability learning and reversal learning.

## Anxiety-related constructs

### Thigmotaxis

*Thigma* originates from the Greek word for touch, and in biology, *taxis* is employed to describe the motion of an organism responding to an external stimulus. The term thigmotaxis refers to the movement of an animal in contact with, or in close proximity to, solid objects, such as maze walls. Rodents, both in nature and in experimental settings, tend to favor movement along the periphery, exhibiting thigmotaxis (Barnett, 1963). This behavioral pattern is considered to be a part of the instinctive defensive repertoire of animals to protect themselves from predators (Barnett, 1963; Grossen & Kelley, 1972). In behavioral testing of rodents, thigmotaxis began to be examined as a stress-driven behavior, following a study that demonstrated an increase in thigmotaxis behavior in rodents exposed to foot shocks (Grossen & Kelley, 1972). Researchers began interpreting thigmotaxis as an indicator of anxiety, as anxiogenic drugs were observed to elevate it, while anxiolytics tended to reduce it (Treit & Fundytus, 1988). These results led to the popularity of employing innate defensive behaviors as indicators of anxiety (Treit, 1985).

#### Open field test

The open field test (OFT) is one of the earliest behavioral tests for rodents, dating back to the 1930s (Hall & Ballachey, 1932). The simple procedure involves placing a rodent into a novel enclosed arena, usually a square-shaped box, and observing its behavior at least for 5 min (refer to Seibenhener & Wooten, 2015 for a video protocol) (Fig. 1). Initially employed to assess the timidity of rodents based on their defecation during the test (Hall, 1934), simplicity of the test led to its widespread adoption. Over time, it became a versatile tool for evaluating not only fearfulness, but also traits relating to exploration, emotionality, and anxiety-like behavior (Prut & Belzung, 2003). A variety of dependent variables can be measured in an open field from rearing counts to urination amounts (Walsh & Cummins, 1976), whereas the time spent in the periphery of the maze (i.e., thigmotaxis) versus its center is used to assess anxiety-like behavior (Seibenhener & Wooten, 2015). The OFT is commonly applied as a control measure in other behavioral tests, such as the FST and TST, to assess potential alterations in general locomotor activity levels of the animals (Gould et al., 2009). As the OFT is used both to assess anxiety-like behavior and measure general locomotor activity, careful consideration is required to determine which dependent variable should be prioritized in each test. This highlights the importance of complementing the OFT findings with supplementary behavioral tests. Additional behavioral monitoring and morphological analysis methods (refer to the dedicated section), such as home cage monitoring, can help to differentiate the locomotor activity of the animal in a nonstressful environment. Furthermore, conducting ultrasonic vocalization (USV) analysis in the OFT can be used to discern the emotional state of the animal (Stanford, 2007).

The behaviors exhibited in the OFT can be perceived as a conflict between two fundamental driving forces: the exploratory drive and the defensive drive (Barnett, 1963). The OFT creates a stressful situation for the animal, not only because the animal is separated from its home cage but also because the test arena is substantially larger (Prut & Belzung, 2003). Within this context, some animals tend to move toward the maze walls and display thigmotaxis. However, the test does not involve any specific aversive stimulus, but a novel environment that may prompt anticipation of potential threats. For this reason, thigmotaxis in the OFT can be interpreted as an indication of anxiety-like behavior, rather than an elicited fear response (Davis et al., 2010) (refer to the Elevated Plus Maze** -** Elevated Zero Maze section for a detailed discussion of the measured constructs in the unconditioned anxiety tests).

In their seminal review, Prut & Belzung (2003) analyzed the effects of different types of anxiolytic drugs in the OFT and concluded that the test is only sensitive to ﻿benzodiazepines and 5-HT1A receptor agonists, offering limited predictive value for other anxiolytics. Several types of medicine that are effectively used in clinical practice to treat disorders associated with different types of anxiety, such as panic attacks and PTSD, did not alter animal behavior in the OFT. Prut & Belzung (2003) concluded that the OFT cannot assess anxiety disorders but still measure normal (levels of) anxiety. As such, spending more time near the maze walls and displaying thigmotaxis was perceived as a rodent form of normal anxiety. The straightforward design of the OFT, however, makes this paradigm more susceptible to variations in external stimuli, such as the lighting conditions of the testing environment (Walsh & Cummins, 1976). Compared with other tests that involve more salient stressors, the baseline conditions of the OFT may produce higher interlaboratory variability in the test results (Schulz et al., 2023).

A recent approach to assess the construct validity of animal models is to do “reverse translation” and directly apply rodent tests to human by using virtual reality (Table 1) (Gromer et al., 2021). To this end, a virtual city walk task, the human analogue of the OFT, revealed that participants with agoraphobia or with high sensitivity scores to anxiety showed greater thigmotaxis compared with the control group (Walz et al., 2016). This study did not only show that the OFT is a human-sensitive task, but it also suggested a novel way to measure anxiety in humans, which often is assessed by self-reports (Grillon & Ernst, 2016). In another virtual reality study, participants exhibited tendency to prefer the peripheral region of an open area irrespective of their trait anxiety (Gromer et al., 2021). This suggests a weak relation between trait anxiety and open-space avoidance in humans, while the relationship between human state anxiety and thigmotaxis awaits to be tested.

### Approach-avoidance conflict

The approach-avoidance conflict arises when an external stimulus has the potential to exert both aversive and rewarding consequences. It triggers conflicting drives, compelling the organism to determine whether to approach or avoid it (Miller, 1952). Humans and other animals evaluate the risks and benefits within their surroundings to employ calculated judgments to choose between approach and avoidance. Neuropsychiatric disorders may disrupt and bias this process toward one side of the conflict. Anxiety disorders often lead to avoidance behavior, which worsens the persistence of the condition (American Psychiatric Association, 2013). Avoidance behaviors vary depending on the type of anxiety. Individuals with social anxiety disorder, for instance, tend to shun social interactions, whereas those with PTSD avoid stimuli connected to their traumatic experiences (American Psychiatric Association, 2013).

The behavioral outcomes of the approach-avoidance conflict are compared between the experimental and control groups to assess anxiety-like behavior in animal models. The maze environment typically serves as the source of approach-avoidance conflict (Montgomery & Monkman, 1955). The subject must decide between exploring the novel test environment for potential rewards, such as food or conspecifics, and retreating to evade potential dangers, such as predators. Exploring relatively bright, open spaces constitutes approach behavior, while remaining in dark, enclosed areas constitutes avoiding (Carobrez & Bertoglio, 2005). This phenomenon is observed in most animals, including primitive species (Schneirla, 1959).

Different behavioral tests are used to assess approach–avoidance conflict in rodents and compare their anxiety-like behavior (Fig. 1). The open field test serves this purpose when its center and periphery are virtually divided into two compartments (Prut & Belzung, 2003). The light-dark box test (LDB) (La-Vu et al., 2020), the elevated plus maze (EPM) (Rodgers & Dalvi, 1997), or the elevated zero maze (EZM) (Shepherd et al., 1994) all provide bright/open and dark/closed areas to elicit approach-avoidance conflict in a spontaneous, unconditioned manner. The Geller-Seifter conflict test (Geller et al., 1962) and the Vogel conflict test (Vogel et al., 1971), in contrast, use conditioned behaviors to assess approach-avoidance conflict. In both of these tests, rodents are trained to associate a food reward (Geller-Seifter conflict test) or water (Vogel conflict test) with a mild electric shock, creating a conflict between their natural approach drives and conditioned avoidance response to these rewarding stimuli (Millan & Brocco, 2003). These two tests are rarely used because of their demanding protocols and limited sensitivity to anxiolytic drugs (Harro, 2018). As an alternative, the novelty-suppressed feeding test (Samuels & Hen, 2011), also utilized in depression studies, combines a food reward with novelty stress to assess the approach behavior in rodents. Finally, social approach-avoidance behavior is used to assess anxiety-like behavior in rodents via social interaction and social approach-avoidance tests (Toth & Neumann, 2013).

The translational relevance of the approach-avoidance conflict is relatively well-studied (refer to Kirlic et al., 2017 for a review). Following the aforementioned reverse translational method, researchers developed a human approach-avoidance task, and revealed that anxiety sensitivity scores are negatively correlated with approach behavior in males, whereas behavioral activation scores are positively correlated with approach for females (Aupperle et al., 2011). Also, neurobiological evidence shows that the homologous parts of the hippocampus, the ventral hippocampus in rodents and anterior hippocampus in humans, play an important role in approach-avoidance conflict in both species (Ito & Lee, 2016).

#### Light-dark box test

The light-dark box (LDB) test was designed to assess the effects of benzodiazepines in the approach-avoidance paradigm (Crawley & Goodwin, 1980). The testing apparatus consists of two compartments, or boxes, connected with a small passage. There is a large, well-illuminated box and a smaller, dark box that is half the size of the illuminated one. The original hypothesis posited that the frequency of the animal's movement between the boxes would indicate approach behavior (Crawley & Goodwin, 1980). This behavior was highly affected by the overall locomotor activity levels of the animals and the testing time-of-day. In following years, the time spent in the dark box started to be associated with anxiety (Bilkei-Gorzó et al., 1998), whereas preferring the light box would reflect an anxiolytic effect (Bourin & Hascoët, 2003; Costall et al., 1989).

The light-dark box test, along with the elevated plus and zero mazes, relies on the innate and unconditioned inclination of rodents to avoid illuminated areas while simultaneously expressing a natural predisposition for exploring novel environments (Bourin et al., 2007; Kumar et al., 2013). In the EPM and EZM, the elevation increases the approach-avoidance conflict for rodents (Pellow et al., 1985; Pellow & File, 1986), distinguishing it from the LDB test. Given that all these tests share a common theoretical foundation as measures of anxiety, the concerns associated with the underlying construct also are shared. Therefore, these issues will be addressed in the following EPM-EZM section. The same considerations apply to the OFT, because it also is employed to assess anxiety by comparing the time spent in the relatively darker peripheral area versus the more illuminated center area (Ennaceur, 2014).

#### Elevated plus maze - Elevated zero maze

The elevated plus maze (EPM) is a cross-shaped apparatus with four arms, typically situated 50 cm above the floor. Two opposing arms are enclosed by opaque walls, whereas the other two arms are open (Pellow et al., 1985). The length and width of the arms and the height of the arm walls differ for rats and mice (Walf & Frye, 2007). The EPM also exploits the approach-avoidance conflict through its dark, enclosed arms (Montgomery, 1955; Montgomery & Monkman, 1955). Testing procedure consists of the placement of a rodent in the center of the maze and observing its behavior and locomotor activity (refer to Komada et al., 2008 for the video protocol). Time spent in the closed versus open arms and the frequency of entries into the closed versus open arms are interpreted as anxiety-like behavior.

Providing enclosed, darker spaces for animals constitute the main trigger mechanism as in the light-dark box test. The EPM uses height to induce additional stress in the open arms, on which the animals perceive that they are situated above the ground but cannot jump off the maze to escape. The open arms of the EPM can be enclosed with transparent walls to prevent accidental falling. In this case, it must be ensured that the illumination in open arms are substantially more than the closed arms (refer to Akmese et al., 2023 for an example). However, enclosing the open arms with acrylic transparent walls diverts from the standard procedure and may reduce the replicability of the findings.

The elevated zero maze (EZM) is a modified version of the EPM, consisting of an annular apparatus divided into two opposite open quadrants and two enclosed quadrants (Shepherd et al., 1994). This maze lacks a center, eliminating the need to analyze and interpret the time spent in the central area, which was a main concern in the EPM. On the one hand, time spent in the center of the EPM can be interpreted as a mild anxiolytic effect (Shepherd et al., 1994). The elevated zero maze removes this option and forces the animals to choose between open or closed areas. This modified test was developed to increase the sensitivity of the construct to a broader range of anxiolytic drugs (Shepherd et al., 1994). Subsequent studies comparing the EPM with the EZM yielded conflicting findings, with some indicating enhanced sensitivity in the elevated zero maze (Kulkarni et al., 2007), whereas others did not (Braun et al., 2011). An additional benefit of the zero maze design is averting behavioral asymmetry that could predispose animals toward a specific direction in the EPM (Schwarting & Borta, 2005). This asymmetry is manifested in the paw preference of rats and has been linked to spatial memory performance and behavioral despair (Ecevitoglu et al., 2020).

Mazes similar to the EPM have originally been used to assess fear-motivated behavior (Handley & Mithani, 1984). The EPM was subsequently conceptualized as an anxiety measure based on the observation that anxiolytics and anxiogenics significantly alter the time spent in open versus closed arms and the number of arm entries (Cruz et al., 1994; Pellow & File, 1986). However, the validity of unconditioned anxiety tests has been questioned, with concerns raised regarding whether the construct being measured corresponds more closely to anxiety, fear-induced escape, or avoidance (Ennaceur, 2014). This critique highlights the theoretical distinction between fear and anxiety—two fundamental concepts that lack consensus in terms of their definition and differentiation within the literature. Earlier perspectives tended to characterize fear as a normal, adaptive response to an immediate threat, whereas fear and anxiety disorders have been viewed as exaggerated, pathological forms of fear responses (LeDoux, 1998; Rosen & Schulkin, 1998). A more recent and widely accepted view focuses on the properties of the stimulus and the range of responses it elicits. According to this perspective, fear arises in response to a known, explicit, or imminent threat, accompanied by active avoidance; whereas anxiety stems from the perception of potential future threats (Barlow, 2000; La-Vu et al., 2020; Perusini & Fanselow, 2015; Robinson et al., 2019; Steimer, 2002), predominantly manifesting with passive avoidance behavior coupled with risk assessment (Kumar et al., 2013; McNaughton & Corr, 2004). The Research Domain Criteria (RDoC) classification also adopts a similar distinction between acute threat (i.e., fear) and potential threat (i.e., anxiety). However, the question of whether the novel environment presented in the aforementioned tests is perceived as an imminent threat or a potential threat, triggering anxiety, continues to be a major point of discussion in the literature (Ennaceur, 2014; La-Vu et al., 2020).

Another concern regarding the constructs assessed in unconditioned anxiety tests underscores the difficulty of establishing a direct correlation between the duration spent in open areas and a reduction in anxiety-like states. This is because heightened exploration tendencies may result in the same observable behavioral outcome (Cryan & Holmes, 2005). To address this limitation, novelty-seeking behavior can be measured with additional assays alongside the aforementioned tests. A related criticism emphasizes that rodents inherently favor dark and confined spaces, and the current tests may not induce a true conflict between their inherent aversion to open spaces and inclination towards exploration (Ennaceur, 2014). In contrast, conditioned anxiety tests, such as the Geller-Seifter conflict test and the Vogel conflict test, more explicitly present the approach-avoidance conflict. The novelty-suppressed feeding test (explained in the next section) introduces this conflict not through conditioning, but by utilizing the hunger drive.

In a more recent study, a mixed reality EPM test has been developed for human subjects, in which the behavioral responses were correlated with both subjective and physiological anxiety measures (Table 1) (Biedermann et al., 2017). In this task, acrophobic fear was correlated with avoidance tendencies regarding the open arms, whereas sensation-seeking traits were linked to an inclination to approach them. However, trait anxiety measures did not show an association with this task. These findings highlight the importance of considering fear and novelty-seeking tendencies as well as state versus trait anxiety differentiation when interpreting the outcomes of unconditioned anxiety tests, such as the EPM. Indeed, these tests are criticized to measure state anxiety, which is a transient construct, rather than the trait anxiety, which is longer in duration and generally considered more related to the anxiety disorders (Andreatini & Bacellar, 2000; Fonio et al., 2012; Markou et al., 2009).

#### Novelty-suppressed feeding test

Reduced eating and defecation reflect the emotional state of rats especially under novelty stress (Hall, 1934). Hyponeophagia, defined as a reduction in feeding behavior in response to novelty, has been documented in both wild and laboratory rat strains (Mitchell, 1976). The novelty-suppressed feeding test (NSFT) (rat protocol: Blasco-Serra et al., 2017; mouse protocol: Samuels & Hen, 2011) evaluates anxiety-like rodent behaviors by examining the conflict between novelty stress and the instinctual urge for feeding (Commissaris, 1993). Hyponeophagia can be induced by introducing novelty in various aspects of feeding behavior, including the food itself (Poschel, 1971), the food container (Mitchell, 1976), or the feeding environment (Hall, 1934). The NSFT protocol includes placing a food-deprived animal to a novel testing arena, in which a small amount of food is located at the center of the well-illuminated maze (Fig. 1). The latency of the animal to approach and bite the food constitutes the dependent variable.

Hyponeophagia-based tests are sensitive to several anxiolytics and chronic antidepressant applications (Dulawa & Hen, 2005). This dual pharmacological response may suggest that chronic antidepressant treatments alleviate anxiety symptoms, as observed in both humans (Bespalov et al., 2009) and other animals (Bodnoff et al., 1988). Alternatively, it may indicate that this test assesses a construct related to both anxiety and depression (Nestler & Hyman, 2010).

#### Social interaction and social approach-avoidance tests

Assessing social interaction levels in animals is widely used to study different psychological disorders, including autism spectrum disorders (Crawley, 2007), schizophrenia (Wilson & Koenig, 2014), and social anxiety disorder (social phobia) (Toth & Neumann, 2013). Social interactions also are used to assess anxiety levels in rodents via a straightforward social interaction test, which involves placing two rats in a designated box and recording their interaction time over a 10-min period (File, 1985; File & Hyde, 1978). Albeit an old procedure, this simple behavioral test retains its relevance in contemporary research, much like the OFT (Acikgoz et al., 2022). Its enduring relevance stems from its lack of requirement for pretraining and avoidance of negative stimuli, such as electrical shocks (File & Seth, 2003). While the social interaction test reflects ethologically natural interactions, it presents challenges in precisely discerning individual levels of social approach or avoidance during these encounters (Harro, 2018; Toth & Neumann, 2013). Subsequent behavioral tests discussed below offer a more controlled approach to measuring an animal’s social approach-avoidance behavior.

The social approach-avoidance test, designed for rats to evaluate stress-induced anxiety (Haller & Bakos, 2002), features a cage divided into a small and a large compartment connected by a tunnel (Fig. 1). An unfamiliar conspecific is placed in the larger compartment behind a perforated, transparent wall, while the test animal is situated in the smaller compartment. Following an acclimation period, the tunnel door is opened, allowing observation of the test animal's entry into the large compartment. Exposure to social defeat stress or electric shocks have been demonstrated to decrease the time spent in the large compartment, an effect mitigated by the anxiolytic chlordiazepoxide (Haller & Bakos, 2002). The sensitivity of this test to both social and nonsocial stressors (i.e., social defeat stress and electric shocks, respectively) highlights its usefulness as a tool for assessing generalized anxiety disorder (Haller et al., 2003). While this test is developed and primarily used with rats, the three-chambered social approach test is more commonly employed in mouse studies (Toth & Neumann, 2013).

The three-chambered social approach test apparatus comprises a central compartment, where the test animal is positioned, and two side compartments adjacent to the center (Landauer & Balster, 1982; Moy et al., 2004; Nadler et al., 2004). One side compartment, the social compartment, contains a conspecific in a wire cage, whereas the other side compartment, the nonsocial compartment, features an empty wire cage (refer to the video protocol: Kaidanovich-Beilin et al., 2011). Following an acclimation period, the rodent freely navigates the maze, and the time spent in the nonsocial compartment and the center serves as an indicator of social avoidance behavior. The test design allows for the comparison of social approach-avoidance behavior towards either a familiar or an unfamiliar conspecific (Moy et al., 2004). In the social novelty preference design, an unfamiliar mouse is introduced into the opposite compartment after the test animal becomes acquainted with the initially present mouse (Moy et al., 2004; Nadler et al., 2004). The original test was later adapted for use with rats (Wee et al., 1995), yet it remained predominantly utilized in mouse studies. Its popularity as a tool for investigating autism spectrum disorder (Moy et al., 2004; Nadler et al., 2004), rather than anxiety, can be attributed to the prevalence of genetic autism models in mice (Wöhr & Scattoni, 2013).

In social interaction and approach-avoidance tests, it is recommended to analyze nuanced variables in the test maze, such as body posture (Jabarin et al., 2022), and incorporate automated tracking in the home cage (Kondrakiewicz et al., 2019; Zilkha et al., 2016) to gain a more precise understanding of the construct under measurement. The behaviors of nonsubject animals can introduce variability or noise in social interaction data. In contrast to social interaction tests that utilize an open field, social approach–avoidance tests address this issue by placing nonsubject animals behind a transparent perforated wall or in a wire cage. Although this setup may not perfectly replicate ecological conditions for social interactions, it successfully minimizes aggressive or sexual interactions between animals, while still enabling the subject animal to approach the non-subject animal (Jabarin et al., 2022). Notably, human studies utilizing virtual reality systems to simulate social interaction scenarios yield consistent results with rodent experiments (Table 1) (Lange & Pauli, 2019; Wieser et al., 2010). These investigations also examine the participants’ morphological features, including facial expressions, gaze, head movements, and body posture—a practice similarly endorsed in rodent studies, as explained below.

## Behavioral monitoring and morphological analysis methods

### Facial expression analysis

Humans typically convey their emotions through changes in their facial muscles, commonly known as facial expressions (Dimberg et al., 2002). Facial expressions corresponding to the six basic emotions (i.e., happiness, disgust, surprise, sadness, anger, and fear) have been observed to be culturally universal (Ekman, 1973; 1993) and are suggested to have biological origins with evolutionarily adaptive functions (Ekman, 1973; 1989). The idea that nonhuman animals also communicate their emotions through facial expressions has been a prevailing notion since Darwin (Darwin, 1872; Ekman, 1973; Waller & Micheletta, 2013). In rodent behavioral testing, the analysis of mimetic elements, specifically changes in the orofacial musculature, was initially used in taste reactivity tests (Grill & Norgren, 1978). This early method involved meticulous, frame-by-frame analysis of video recordings. Subsequently, pain researchers began to utilize facial expressions as an indicator of discomfort and devised a quantification system known as the mouse grimace scale (Langford et al., 2010). When this scale was adapted to rats, a semiautomated software tool was developed to identify and categorize rat facial expressions from video recordings (Sotocinal et al., 2011).

Inferring taste and pain related states through analyzing particular muscle movements in the rodent face made it apparent that the use of this technique can go beyond human experiments. The analysis of facial expressions soon emerged as a valuable tool for assessing the affective states of rodents, encompassing both negative (Defensor et al., 2012) and positive states (Finlayson et al., 2016). The availability of automated analysis tools that utilize artificial intelligence (Isik & Unal, 2023) transformed this labor-intensive technique to a relatively straightforward and reliable task. In a recent study, facial expressions of mice during exposure to aversive or rewarding stimuli were automatically detected and categorized via use of machine learning (Dolensek et al., 2020). Furthermore, with reverse engineering, an algorithm was trained to predict the emotional state of the animal from its facial expressions. This study elucidated the basic properties of emotions, such as intensity, valence, flexibility, generalization, and persistence, across various test designs. It also elucidated the distinct neural underpinnings of clustered emotions through the use of optogenetics. Altogether, these investigations underscore the potential of facial expression analysis in the domain of animal emotion research.

### Ultrasonic Vocalization (USV) analysis

Several species use vocal signals to communicate with conspecifics, especially to produce mating calls and alarm signals (Fichtel & Manser, 2010). Rodents communicate via emitting sounds in the ultrasonic range (>20 kHz) (Faure et al., 2017; Simola & Brudzynski, 2018), which is beyond the auditory spectrum of human perception. Rodents live in social groups in nature (Barnett, 1963), and using signals within the ultrasonic range enable them to communicate without attracting the attention of potential predators (Simola & Brudzynski, 2018). Different rodent species emit distinct types of vocalizations depending on their age and the context (refer to Portfors, 2007 for a review). The most extensively studied rodent vocalizations include maternal separation signals of pups (Olivier et al., 1998; Wöhr & Schwarting, 2008), signals emitted during juvenile play interactions (Burke et al., 2018), and adult vocalizations occurring in social contexts, including signals for mating (McGinnis & Vakulenko, 2003) and aggression (Thomas et al., 1983).

Rat USVs can be categorized into two major groups in terms of the affective state they represent: signals associated with appetitive stimuli (≈50 kHz) and signals linked to aversive conditions (≈22 kHz) (Wöhr & Schwarting, 2013). In rodent research, USV recordings are also used to manipulate the affective state of the animals by replaying USV recordings back to them (Niemczura et al., 2020). The playback of 50-kHz signals to rats induces approach behaviors (Wöhr & Schwarting, 2007), whereas 22-kHz USVs produce the opposite effect, inducing avoidance responses (Brudzynski & Chiu, 1995). Similar to facial expression analysis, different machine learning methods are increasingly applied to automated classification of rodent USV signals. Supervised learning often is used for automatic detection of preestablished categories (Fonseca et al., 2021; Premoli et al., 2021; Vogel et al., 2019), whereas unsupervised learning methods are utilized to create, detect, and form USV clusters that may extend beyond the capacity of human expertise (Coffey et al., 2019; Van Segbroeck et al., 2017). Ultrasonic vocalizations are generally recorded in a separate soundproof test apparatus during behavioral testing in order to separate them from the background noise. Recently, a protocol was developed to acquire and isolate USVs within the home cage in conjunction with other home cage monitoring methods (Hobson et al., 2020).

### Posture analysis

Examining the body posture of an animal provides a useful tool for gaining insights into its overall well-being. For instance, a rodent in pain often exhibits a characteristic hunched back posture (Carstens & Moberg, 2000). During conflicts with conspecifics and physical encounter with predators, rodents may adopt defensive or threatening postures that reflect their tendency to fight or flight (Barnett, 1963; Blanchard et al., 1977). The body posture can serve as an indicator of specific behavioral patterns in the context of animal testing. An illustrative example is the stretched attend posture (SAP), where the animal remains motionless while stretching its upper body to explore and sniff a new area. This behavior is considered a manifestation of risk assessment (Riebe & Wotjak, 2012) and often is observed in anxiety-related tests based on exploratory drives, such as the EPM (Espejo, 1997). Moreover, it has been demonstrated that the SAP is responsive to several anxiolytic drugs (Molewijk et al., 1995). A software tool has been developed for the automatic detection and analysis of the SAP to be used in the OFT and EPM as an additional measure (Holly et al., 2016).

Detecting and categorizing body postures during stable moments or execution of a particular behavior yields valuable insights into the constructs associated with a particular symptom. For instance, in the EPM, one might infer that the animal is experiencing a low level of anxiety when more than half of its body is located in the open arm. However, a closer examination might reveal that the animal is mainly exhibiting SAP and exploring the maze without leaving its perceived safe zone, the closed arm. Recognizing the potential of posture analysis and employing machine learning techniques to analyze posture data across various tests has been proposed as a “pose-tracking revolution” (von Ziegler et al., 2020). Many open-source, AI-based software has been developed in recent years to track rodents during behavioral testing, perform pose estimation, and categorize their behaviors (refer to Isik & Unal, 2023 for a review). These novel tools did not only facilitate and accelerate behavioral analysis, but also unveiled micro-behavioral patterns that were otherwise unnoticeable to the naked human eye during manual analysis. These microbehavioral patterns are referred as behavioral syllables (Wiltschko et al., 2020). Identifying new behavioral syllables that are associated with particular rodent constructs may strengthen the construct validity of behavioral tests by providing additional behavioral markers.

### Home cage monitoring

The behavioral tests described in this review entail moving the animal from its home cage to an experimental apparatus for a brief amount of time. This typical procedure of placing the animal to the test apparatus is susceptible to multiple factors including the animal's adaptation to a novel environment and its level of interaction with the experimenters (Chesler et al., 2002). In addition, although rats and mice are nocturnal animals, behavioral tests are conventionally conducted during daytime. These factors can interfere with the results of behavioral testing. To address these concerns, home cage monitoring (HCM) systems have been developed to measure animals’ behavior within the cage they live in, without human intervention or environmental alterations (Grieco et al., 2021; Klein et al., 2022; Mingrone et al., 2020). These systems allow for continuous monitoring, including nighttime (i.e., after the vivarium lights are turned off) when the rodents are substantially more active.

The home cage monitoring systems were designed primarily for tracking an animal’s locomotor activity. These techniques involve either capturing the electrical changes in the cage generated by the animal’s movements through electrodes positioned under the cage (Iannello, 2019) or monitoring specific animals via radio frequency identification (RFID) tags affixed to them (Kiryk et al., 2020). However, these early systems only record the animals’ total travel distance and speed within the cage and do not facilitate the classification of behaviors. In other systems, in addition to locomotor activity, certain behaviors can be classified by using infrared beam alterations surrounding the cage (Brown et al., 2016) or mechanical vibration sensors located beneath the cage (Van De Weerd et al., 2001). Unless integrated with the explained morphological analysis methods, home cage monitoring systems do not offer the capability to detect and categorize specific behaviors.

Behavioral analyses in the home cage require employing pose tracking tools either in real-time (online) or offline on recorded video (Jhuang et al., 2010). It is important to note that the video-based methods may not be optimal for the simultaneous analysis of multiple animals, particularly when there are no distinct visual features that differentiate individual animals. A potential solution to this challenge involves integrating radio wave tracking of tagged animals with video analysis techniques, thereby enabling the investigation of social behaviors exhibited by animals in their niche (Bains et al., 2018; Peleh et al., 2019).

In addition to enabling the observation of rodent behaviors within their home cage, these systems offer the capability to transform the home cage into an operant chamber or a testing apparatus. By integrating wall-mounted nose pokes or levers to observe an animal's behavior and dispense food or drink as a reward for specific actions, it becomes possible to train and assess animals within their home cage (Balzani et al., 2018). Furthermore, manipulating the home cage provides a method for conducting anxiety tests based on approach-avoidance conflicts. A specific test, referred to as the light spot test or the PhenoTyper test (named after a private company), entails illuminating a specified area of the home cage’s food dispenser during the initial phase of the dark period when the animal is most active (Aarts et al., 2015). Researchers showed that the introduction of an active light spot led to a decrease in the time mice spent outside their shelter, and this effect was mitigated by the administration of the anxiolytic, diazepam. The adaptation of the light spot test to rats produced consistent findings, confirming its reliability as a measure of avoidance behavior or anxiety (Kyriakou et al., 2018). It alleviates the impact of experiment-related factors, such as handling (Henderson, Dani et al., 2020a, 2020b; Henderson, Smulders et al., 2020a, 2020b) before the test and the novelty of the test environment. Notably, the light spot test enables researchers to make direct comparisons of animal behaviors before, during, and after manipulative interventions (Prevot et al., 2019). These comparisons revealed that the avoidance response elicited by light persists for hours after the light is no longer present, termed residual avoidance, and can extend up to 6 weeks (Prevot et al., 2019). These findings show that both acute and long-term avoidance behavior can be assessed through home cage monitoring.

A compelling demonstration of the efficacy of home cage monitoring in the field of affective disorder research is illustrated by the work of Goodwill et al. (2019), who identified indicators of depression in female mice subjected to early life stress. Notably, these indicators are symptoms analogous to those observed in human depression, encompassing changes in sleep patterns, lethargy in walking, and reduced self-care manifested as a decrease in grooming. In this study (Goodwill et al., 2019), female mice subjected to early life stress exhibited higher levels of immobility in the FST, displaying a sex-specific pattern. This observation highlights the value of long-term behavioral measurements in mitigating concerns related to estrous cycle variations, a major factor contributing to the underrepresentation of female subjects in behavioral studies. Hence, home cage monitoring can substantially facilitate the study of sex differences in affective disorders. These systems have demonstrated success in evaluating the effectiveness of both conventional (fluoxetine) (Alboni et al., 2015) and rapid-acting (i.e., ketamine) (Goodwill et al., 2019) antidepressants.

In summary, home cage monitoring techniques yield favorable outcomes by ensuring the accurate measurement of targeted constructs, eliminating the inherent variability of novel testing environments, and enabling the assessment of behaviors that may not manifest within brief timeframes or under unfamiliar conditions. However, a persistent challenge in the field is the absence of a valid, ground truth measure against which to assess home cage behaviors. The question always arises about how specifically the relevance of a behavioral change is linked to an affective state rather than other effects, such as changes in motivation or apathy.

## Conclusions

Contemporary neuroscience has witnessed significant methodological advancements that expanded our understanding of the neurobiological, electrophysiological, and neuroanatomical properties of the brain (Bassett & Sporns, 2017; Deisseroth, 2011; Wilt et al., 2009). Yet, behavioral testing in rodents, an age-old technique, continues to serve as the primary method for providing a theoretical framework to interpret these findings (Krakauer et al., 2017). Identifying the behavioral features and outcomes of neurobiological and neuropsychiatric phenomena is essential for achieving a comprehensive understanding of the system under investigation. However, when the constructs assessed in behavioral tests are not critically examined, these assessments become instruments that merely reveal the impacts of specific drugs without clarity on what they are actually measuring at a broader cognitive and affective level (Rodgers et al., 1997).

This review explored behavioral constructs and rodent tests associated with clinical depression and anxiety. Theoretical origins and the evolution of each construct were discussed, along with an examination of the test protocols employed for assessing these constructs. Specific concerns about the construct validity of these behavioral tests were addressed, highlighting the limitations of the historically dominant, symptom-based interpretation. Instead, a new perspective was introduced through the RDoC framework (Sanislow et al., 2010), which deconstructs the symptom-based higher order constructs into simple behavioral patterns, establishing a common theoretical ground between behavioral testing and human psychopathology. The current understanding is supported by protocols that aim to measure the same construct in both humans and rodents. Through reverse translation, many traditional rodent tests have been adjusted for use in humans, whether in real or virtual environments (Table 1). Interestingly, the recent cognitive affective bias measurements take the opposite approach, adapting human constructs and measurement methods for use in rodents.

Finally, supplementary behavioral monitoring and morphological analysis methods that are applicable in both rodents and humans, such as facial expression and posture analysis, hold the potential to enhance the theoretical connection between behavioral constructs evaluated in rodents and human psychopathology. Other monitoring techniques, such as USV recordings and home cage monitoring concentrate on species-typical rodent behaviors. Overall, integrating these additional monitoring methods into behavioral testing can significantly enhance the interpretation of the construct validity of the discussed behavioral tests.
